# Supplementary material for: Competitive capabilities of higher education institutions from their Employees’ perspectives: A case study of King Khalid University
Source: PLoS One. 2024 May 6;19(5):e0302887. doi: 10.1371/journal.pone.0302887 (PMC11073671; doi:10.1371/journal.pone.0302887)
Supplement: Attachment — Submitted filename: Competitive Capabilities KKU.docx [file pone.0302887.s001.docx]

**Title: Competitive Capabilities of Higher Education Institutions: A Case Study of King Khalid University (KKU)**

The title of the abstract is specific to a certain institution making it more localized. Besides, two authors affiliated with the university where the research took place can affect the results.

**Abstract:**

The KKU first mentioned in the abstract should be written in full.

The first sentence in the abstract KKU’s competitive strength in what?

The fourth sentence on the results revealed that ………………… is too long and should be restructured.

Thematic analysis results revealed that……….. the use of thematic analysis was becoming more common in the abstract.

**Introduction**

After the introduction, the sub-headings that came after should be well-known to the audience as to whether they are literature reviews or not. Again, I was expecting that you say something about KKUs in terms of the institutions' competitiveness with other institutions and the need for this study briefly. This will inform readers what is expected to follow in subsequent sections of the work.

Methodology

Method

The first sentence is it depth interviews or in-depth interviews?

Participants

How do you ensure that there were no biases in the study as two of the participants were from the university?

**Discussion**

The first paragraph of the discussion should state the key findings of the study. This should be followed by subsequent sections trying to link the findings with previous literature.
